# Mucosal incision combined with balloon dilation as a useful method for anastomotic stricture with ulcer

**DOI:** 10.1055/a-2686-3487

**Published:** 2025-09-09

**Authors:** Keisaku Yamada, Masahiro Tajika, Tsutomu Tanaka, Nobuhito Ito, Akihiro Takagi, Yasumasa Niwa

**Affiliations:** 1 Department of Endoscopy, Aichi Cancer Center Hospital, Nagoya, Japan

Benign esophageal stricture may occur as an anastomotic stricture following esophageal surgery or endoscopic submucosal dissection for extensive superficial esophageal cancer. Once an esophageal stricture develops, it often causes dysphagia and requires multiple endoscopic balloon dilation (EBD). Recently, it has been suggested that combining mucosal incision with EBD enables consistent tearing of the esophageal mucosa, reducing the incidence of perforation and improving the rate of restenosis 1 2 .

We report a case in which we used mucosal incision combined with EBD to treat anastomotic stricture with ulcer, which has been considered difficult to treat with conventional EBD.

The patient was a 79-year-old male with anastomotic stricture following esophageal surgery. However, he had ulcers on the stricture at the time of the previous EBD and did not undergo. So, oral treatment with PPI was administered, and EBD was attempted again this time, but the ulcers did not improve ( Fig. 1 ). Therefore, mucosal incision combined with EBD was performed ( Video 1 ).

**Fig. 1:**
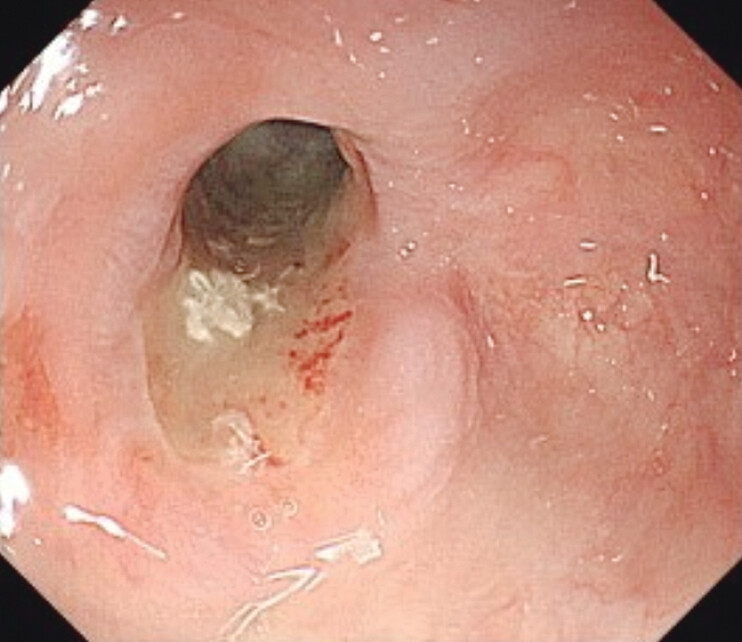
Anastomotic stricture with ulcer was present, and the scope could not pass.

Mucosal incision combined with balloon dilation for anastomotic stricture.Video 1

Anastomotic stricture with a half-circular deep ulcer was observed, and the scope could not pass. At first, three mucosal incisions were made with a needle knife (KD-645L: Olympus), avoiding the deep part of the ulcer ( Fig. 2 ). Then, balloon dilation was performed. After dilation, it was confirmed that there was no perforation, and the balloon was reinserted for re-dilation to 15 mm ( Fig. 3 ). After dilation, it was confirmed that the scope could pass and there was no complication. Furthermore, it was observed that the areas of prior mucosal incisions were consistently dilated, and the procedure was completed ( Fig. 4 ).

**Fig. 2:**
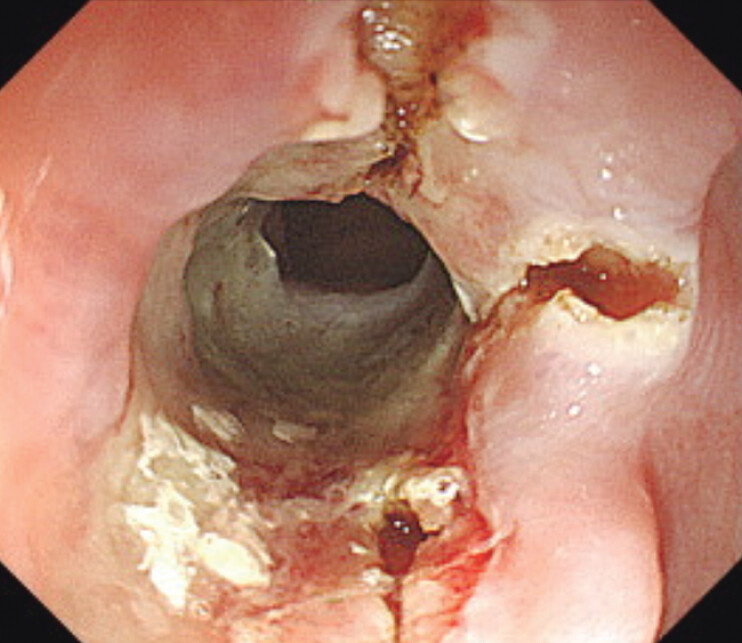
Three mucosal incisions were made with a needle knife, avoiding the deep part of the ulcer.

**Fig. 3:**
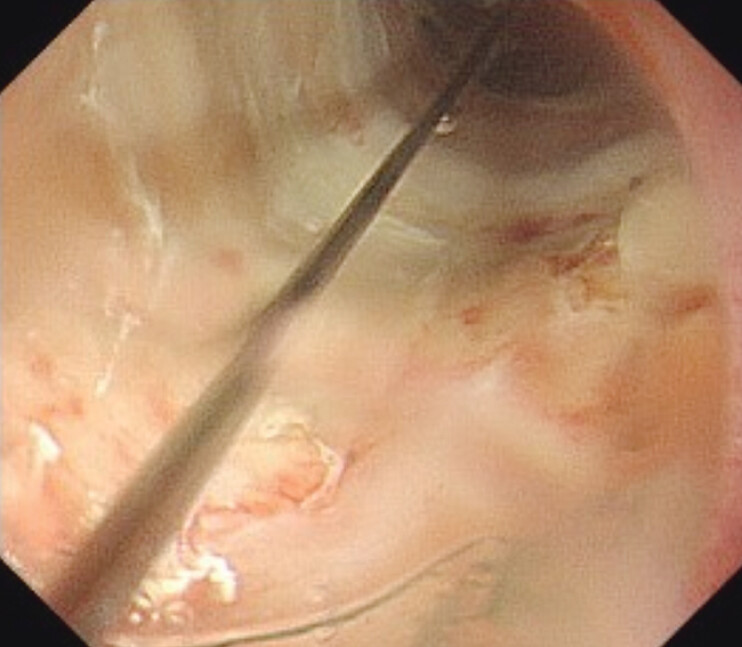
Balloon dilation was performed to 15 mm.

**Fig. 4:**
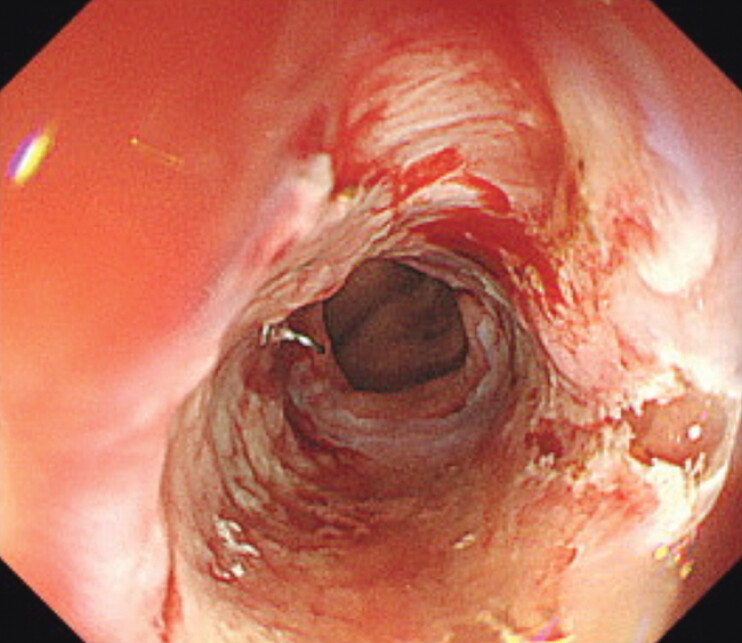
After dilation, the areas of prior mucosal incisions were consistently dilated without perforation.

By performing a mucosal incision prior to EBD, it was possible to control the direction of mucosal tearing and safely expand the stricture without applying excessive force to the ulcer. Combining mucosal incision with EBD is useful for anastomotic stricture with an ulcer.

Endoscopy_UCTN_Code_TTT_1AO_2AH

## Notes

Endoscopy E-Videos https://eref.thieme.de/e-videos
*E-Videos* is an open access online section of the journal *Endoscopy* , reporting on interesting cases and new techniques in gastroenterological endoscopy. All papers include a high-quality video and are published with a Creative Commons CC-BY license. Endoscopy E-Videos qualify for HINARI discounts and waivers and eligibility is automatically checked during the submission process. We grant 100% waivers to articles whose corresponding authors are based in Group A countries and 50% waivers to those who are based in Group B countries as classified by Research4Life (see: https://www.research4life.org/access/eligibility/ ). This section has its own submission website at https://mc.manuscriptcentral.com/e-videos .
